# Association of vitamin D and FGF23 with serum ferritin in hypoparathyroid thalassemia: a case control study

**DOI:** 10.1186/s12882-020-02101-3

**Published:** 2020-11-16

**Authors:** Forough Saki, Azita Salehifar, Seyed Reza Kassaee, Gholamhossein Ranjbar Omrani

**Affiliations:** Shiraz Endocrinology and Metabolism Research Cente, Shiraz University of Medical Sciences, Shiraz, Iran

**Keywords:** PTH, FGF23, Ferritin, L,25(oh)_2_D_3_, Hypoparathyroidism, Major beta-thalassemia

## Abstract

**Background:**

FGF23 controls serum l,25(OH)_2_D_3_ levels and phosphate homeostasis. This study evaluates the effects of ferritin on intact PTH, FGF23, and l,25(OH)_2_D_3_ in patients with major thalassemia. It also evaluates FGF23 changes in patients with hypoparathyroidism to clarify the interaction between FGF23 and PTH in the absence of proper PTH functioning in human.

**Methods:**

In this case-control study, 25 major-beta thalassemia patients with hypoparathyroidism were age- and gender-matched with major-beta thalassemia patients having normal parathyroid function. Biochemical studies assessed the serum calcium, albumin, phosphorus, alkaline phosphatase, PTH, FGF23, 25(OH) D, 1,25(OH)2D3, ferritin, and the fractional excretion of phosphorous.

**Results:**

FGF23 was higher in the patients with hypoparathyroidism than the controls (*P* = 0.002). The fractional excretion of phosphorous was lower in patients with hypoparathyroidism, despite the high level of FGF23 (*P* = 0.001). There was a correlation between serum 1,25(OH)2D3 and FGF23 with ferritin in the controls (*P* = < 0.001and *P* = < 0.001, respectively).

**Conclusions:**

The present study showed a strong positive correlation between serum ferritin and levels of FGF23 and 1,25(OH)2D3. We hypothesized that ferritin could have a stimulatory effect on the production of 1,25(OH)2D3. Moreover, a rise in FGF23 in patients with thalassemia, might be either associated with the stimulating effect of PTH and 1,25(OH)2D3, or directly related to the stimulating effect of ferritin.

## Background

Thalassemia is an inheritable disease caused by abnormal hemoglobin production, resulting in ineffective erythropoiesis and increased peripheral hemolysis. The clinical outcomes of iron overload vary and reflect the key location of iron deposition. The concentration of ferritin in serum provides a quantitative measure for iron storage [1]. In patients with major thalassemia, frequent blood transfusion and iron overload, despite intensive chelation therapy, make them prone to many endocrine complications, such as hypogonadotropic hypogonadism, diabetes mellitus, hypothyroidism, and hypoparathyroidism [2, 3].

PTH is a potent stimulator in producing l, 25(OH)_2_D_3_ by increasing 1-alfa-hydroxylase activity in the proximal renal tubules. Reduced PTH secretion results in hypocalcemia and hyperphosphatemia [4, 5]. PTH and fibroblast growth factor 23 (FGF23) are the primary hormones that regulate the phosphate and calcium homeostasis [6]. FGF23 is a member of FGF19 subfamily, produced by osteocytes in response to high levels of serum phosphate and l,25(OH)_2_D_3_ [7, 8]. FGF23 acts through FGFR-klotho co-receptors in the kidneys to provoke phosphaturia and diminish 1-alfa-hydroxylase activity. It also controls the production of l,25(OH)_2_D_3_ [9–12].

Previous studies have shown the effects of dietary phosphate and serum phosphate on the release of FGF23 [13, 14]. The role of interaction between PTH and FGF23 on the regulation of serum phosphate is not clearly understood. Recent studies have shown that both iron deficiency and iron transfusion exert some effect on the serum FGF23 [15–18]. However, there is a lack of sufficient data evaluating the association or interaction between high serum ferritin and levels of serum l,25(OH)2D3, FGF23 and PTH in patients with thalassemia.

In addition, high and prolonged serum FGF23 has many possible effects in body tissues such as blood, bone and cardiovascular system. It has been shown that elevations in serum FGF23 concentrations positively correlate with increased serum levels of inflammatory markers in patients with CKD. Many clinical studies have reported the associations between FGF23 and inflammatory markers in disease states [19–22]. Another effect of FGF23 on the hematopoietic system was proposed in prior investigation that revealed an inhibitory role of FGF23 red blood cell production was reported. Consequently, anemia occurs in conditions of long-term increase in serum level of FGF23 [23, 24]. Moreover, some experimental data suggest FGF23 is a risk factor that associates with cardiac pathologies and cardiovascular mortality. Prolonged elevations FGF23 induce changes in cardiac morphology and eventually heart failure [25, 26]. Another effect of prolonged rise in serum FGF23 was on bone health. Elevated levels of the FGF23 have been related to lower bone mineral density (BMD) and greater risk of fractures in Patients with thalassemia major [27]. It is possible that in patients with thalassemia, prolonged elevated serum FGF23 could aggravate anemia and prone them to cardiovascular and bone complications.

In our previous case-control study, we evaluated FGF23 function in hypoparathyroid patients compared to a healthy population, and we find that although the FGF23 is a main regulator of urinary phosphate excretion but the existence of sufficient parathyroid hormone is necessary for the full phosphaturic effect of FGF23 [28]. The aim of this study was to evaluate the association of ferritin, intact PTH, FGF23, and l,25(OH)2D3 in patients with major thalassemia having normal parathyroid function and hypoparathyroidism.

## Methods

This study was done on 25 patients with major-beta thalassemia as well as hypoparathyroidism from October 2017 to March 2018 at Shiraz University of Medical Sciences affiliated thalassemia clinics in Fars province, southern Iran. A total of 25 age- and gender-matched participants who had major-beta thalassemia with normal parathyroid function, were selected as the control. At the time of diagnosis, hypoparathyroidism was observed by hypocalcemia (Ca less than 8.5 mg/dl) in spite of having inappropriate low intact parathyroid hormone (iPTH) and high serum phosphate level in thalassemia endocrine clinic. All patients with hypoparathyroidism had routine follow-up by one proficient endocrinologist and treated by appropriate doses of calcium carbonate (500 mg tablet, Toliddaru pharmaceutical, Tehran, Iran), plus calcitriol (0.25 μg capsule, Zahravi Pharmaceuticals, Tehran, Iran). The range of daily dose of calcitriol was 0.5–2.5 μg/day to correct serum calcium [5, 28]. The majority of transfusion-dependent thalassemia patients received routine blood transfusion therapy every 3–4 weeks to maintain their hemoglobin levels at 9–10.5 g/dL. In these patients, iron chelating agents, such as oral chelators (deferasirox and deferiprone) and deferrioxamine injection were used. Deferoxamine subcutaneous injection (20–40 mg/kg/day) was used in thalassemia patients with a serum ferritin level greater than 1000 ng/mL. The exclusion criteria in both groups were renal failure (glomerular filtration rate less than 60 mL/min), liver failure, and other metabolic bone diseases (e.g., rickets), hyperthyroidism, and diabetes mellitus.

Blood samples were obtained from all participants for a minimum of 15 days after transfusion and overnight fasting. All blood samples were centrifuged and the sera were stored at − 70 °C at the Endocrinology and Metabolism Research Center Laboratory of Shiraz University of Medical Sciences. Serum calcium (mg/dL), phosphorus (mg/dL), albumin (g/dL), and alkaline phosphatase (IU/L) levels, were measured by colorimetric assays using an SA auto-analyzer (Biosystems SA, Spain). Electrochemiluminescence methods were used to measure serum parathyroid hormone (PTH) (pg/mL) and 25(OH) D (ng/mL) levels using a Cobas E411 (Roche, Germany). Serum intact FGF23 (pg/mL) and 1,25(OH)2D3 (pg/mL) was analyzed by ELISA method using an ELISA kit, (Bioassay Technology, Spain). Sensitivity, intra- and inter-assay CVs of all kits was the same as our previous published article [28]. Serum ferritin levels were recorded on an E 170 analyzer (Roche Diagnostics, Germany) by Electrochemiluminescence’s Immunoassay (ECLIA) method.

### Ethical statement

This study was approved at Shiraz University of Medical Sciences Local Ethics Committee and Vice-Chancellor of Research at SUMS (approval number:1396-01-01-15,805).

### Statistics

Statistical analysis was performed using SPSS statistical software (SPSS 22, IBM SPSS software, Armonk, NY). Data are shown as mean ± SD. Sample size formula was used to compare the two independent groups (*α* = 0.05, *β* = 0.2) as 25 participants in each group. Kolmogorov– Smirnov test was used to evaluate the normality of data distribution. Comparison of normally distributed data were done using Student’s *t-*test, while that of not-normally distributed ones was compared using Mann–Whitney test. Correlations between normally distributed parameters and non-normally distributed ones was analyzed using Pearson’s test and Spearman’s ranking test, respectively. *P-*values less than 0.05 were considered to be statistically significant. A multiple linear regression model was employed to determine the independent factors influencing FGF23 in both case and control groups. Pearson chi-square test was used to compare different groups of iron chelating agents. Multiple linear regression analysis was also employed to explore the factors determining the serum FGF23 and 1,25(OH)2D3 levels in the control group and patients with hypoparathyroidism.

## Results

In the present study, 50 patients with beta thalassemia including 25 cases with hypoparathyroidism and 25 controls with normal parathyroid function were enrolled. The patients’ mean ages in the case and control groups were 26.9 ± 3.09 years and 25.7 ± 5.1 years, respectively. The hypoparathyroid group included 59.3% male participants. General characteristics and biochemical parameters of the patients are summarized in Table 1. The mean serum calcium and PTH levels were lower in patients with hypoparathyroidism (8.7 ± 1.6 mg/dL, and 13.93 ± 4.6 pg/mL) than the control (10.1 ± 0.9 mg/dL, and 55.6 ± 15.7 pg/mL), (*P* = 0.001 and *P* < 0.001, respectively). Serum phosphorus and FGF23 levels were significantly higher in patients with hypoparathyroidism (5.9 ± 1.6 mg/dL, and 381.9 ± 175 pg/mL) than the control group (4.8 ± 0.8 mg/dL, and 241.2 ± 121.0 pg/mL) (*P* = 0.002 and *P* = 0.005 respectively). The mean FE phosphorous was lower in the case group (5.1 ± 3.1%) than the controls (9.2 ± 5%) (*P* = 0.001). There was no significant difference in serum alkaline phosphatase, 25(OH) D, ferritin, 1,25(OH)2D3, and urine Ca/Cr ratio between the case and control groups (*P* = 0.65, *P* = 0.22, *P* = 0.08, *P* = 0.51, and *P*p = 0.92, respectively).

**Table 1:** General characteristics and biochemical studies in both case and control groups and the related comparisons

Variable	Control	Case	***P*** value
**Age (y)**	25.7 ± 5.1	26.9 ± 3.0	0.32
**Weight (Kg)**	50.5 ± 9.3	54.04 ± 10.2	0.213
**Height (cm)**	157.54 ± 8.832	162.40 ± 10.642	0.089
**BMI (Kg/m**^**2**^**)**	21.4 ± 7.5	20.3 ± 2.4	0.479
**PTH (pg/mL)**	55.6 ± 15.7	13.93 ± 4.6	< 0.001
**Ca (mg/dl)**	10.1 ± 0.9	8.7 ± 1.6	0.001
**Ph (mg/dl)**	4.8 ± 0. 8	5.9 ± 1.6	0.005
**Alk (IU/L)**	279.3 ± 149.0	260.9 ± 121.5	0.65
**1,25(OH)2D3 (pg/mL)**	101.2 ± 38.1	94.7 ± 31.6	0.51
**25 (OH) D (ng/mL)**	21.6 ± 4.5	23.8 ± 7.8	0.22
**FGF23 (pg/mL)**	241.2 ± 121.0	381.9 ± 175.0	0.002
**Ferritin (ng/mL)**	1690 ± 548	1396 ± 638	0.086
**FE phosphorous (%)**	9.2 ± 5.0	5.1 ± 2.9	0.001
**Urine Ca/Cr ratio**	0.16 ± 0.2	0.17 ± 0.1	0.92

*FGF*_*23*_ Fibroblast Growth Factor 23, *Ph* Phosphorus, *Ca* Calcium, *PTH* Parathyroid Hormone, *FEph* Fraction excretion of phosphorus, *P* Predictive value

**Fig. 1:**
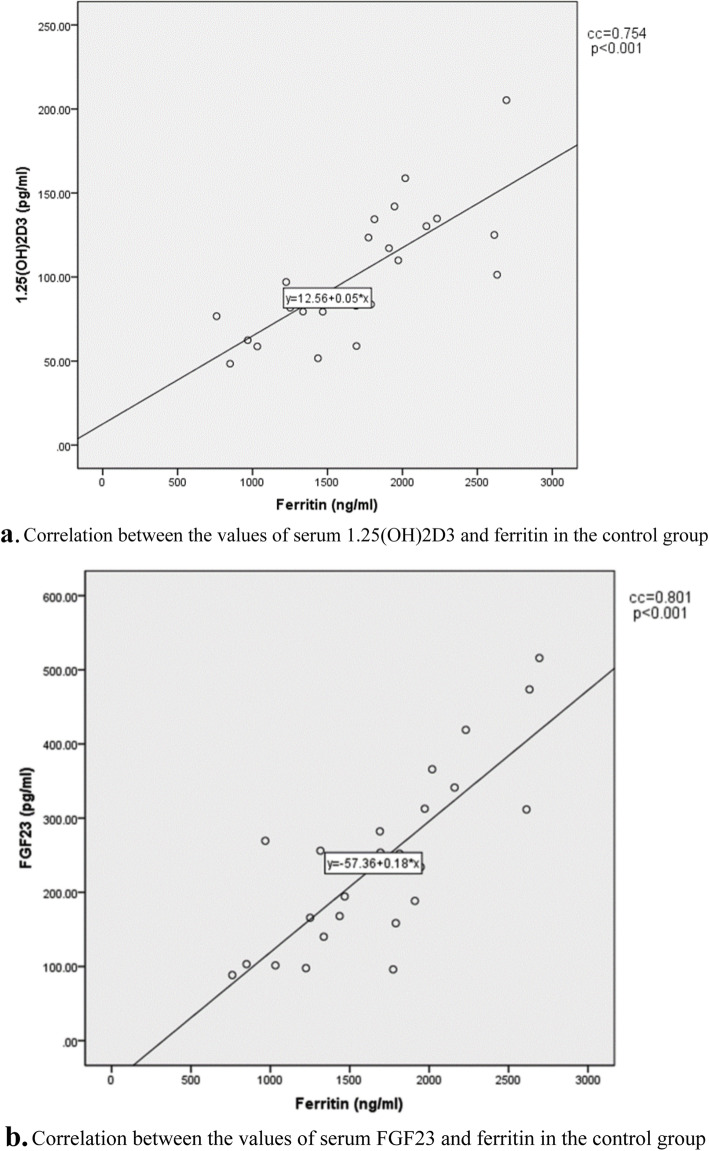
Correlation between the values of serum ferritin and 1.25(OH)2D3 (**a**) and the correlation between the values of serum ferritin and FGF23 (**b**) in the control group

In the control group, there was a positive strong correlation between serum ferritin and FGF23 (*P* < 0.001, CC:0.801), as well as between serum ferritin and 1,25(OH)2D3 (*P* < 0.001, CC:0.754), Fig. 1a and b. However, serum ferritin level in patients with hypoparathyroidism did not correlate with the levels of serum FGF23, calcium, phosphorous, PTH, 25(OH) D, 1,25(OH)2D3, and FEPh (*P* > 0.05). Tables 2 and 3 shows the multiple linear regression analysis of the covariates of FGF23 and 1,25(OH)2D3 in both case and control groups. It shows that association of ferritin with FGF23 or 1,25(OH)2D3 persisted after considering other contributing factors such as serum Ca, Ph, and PTH.

**Table 2:** The multiple linear regression analysis of covariates of 1,25(OH)_2_D_3_ in both case and control groups, performed by enter method

Group	Associated factor	Beta	***P*** value
**Control** **(R square = 0.534)** ***P*** **< 0.001**	Ferritin (ng/mL)	0. 71	0.016
	FGF23 (pg/mL)	− 0.01	0.96
	PTH (pg/mL)	0.21	0.20
	Ca (mg/dL)	0.03	0.84
**Case** **(R square = −0.081)** ***P*** **= 0.72**	Ferritin (ng/mL)	0.08	0.68
	FGF23 (pg/mL)	0.27	0.20
	PTH (pg/mL)	0.036	0.88
	Ca (mg/dL)	0.168	0. 5

*FGF*_*23*_ Fibroblast growth factor 23, *Ca* Calcium, *PTH* Parathyroid hormone

**Table 3:** The multiple linear regression analysis of covariates of FGF23 in both case and control groups, performed by enter method

Group	Associated factor	Beta	***P*** value
**Control** **(R square = 0.651)** ***P*** **< 0.001**	Ferritin (ng/mL)	0. 77	0.001
	1,25(OH)_2_D_3_ (pg/mL)	−0.035	0.86
	PTH (pg/mL)	0.26	0.075
	Ph (mg/dL)	−0.072	0.65
**Case** **(R square = −0.027)** ***P*** **= 0.52**	Ferritin (ng/mL)	−0.17	0.42
	1,25(OH)2D3 (pg/mL)	0.27	0.19
	PTH (pg/mL)	−0.12	0.56
	Ph (mg/dL)	0.136	0. 5

*PTH* Parathyroid hormone, *Ph* Phosphorous

Based on the received iron chelating agents, patients were divided into four groups according to the daily dose of deferrioxamin and deferasirox (group1 < 500 mg/day, group2 > 500–1000 mg/day, group3 > 1000–1500, and group4 > 1500–2000). There was no significant difference in the dosage and kind of iron chelating agents received between the two groups.

## Discussion

**Fig. 2:**
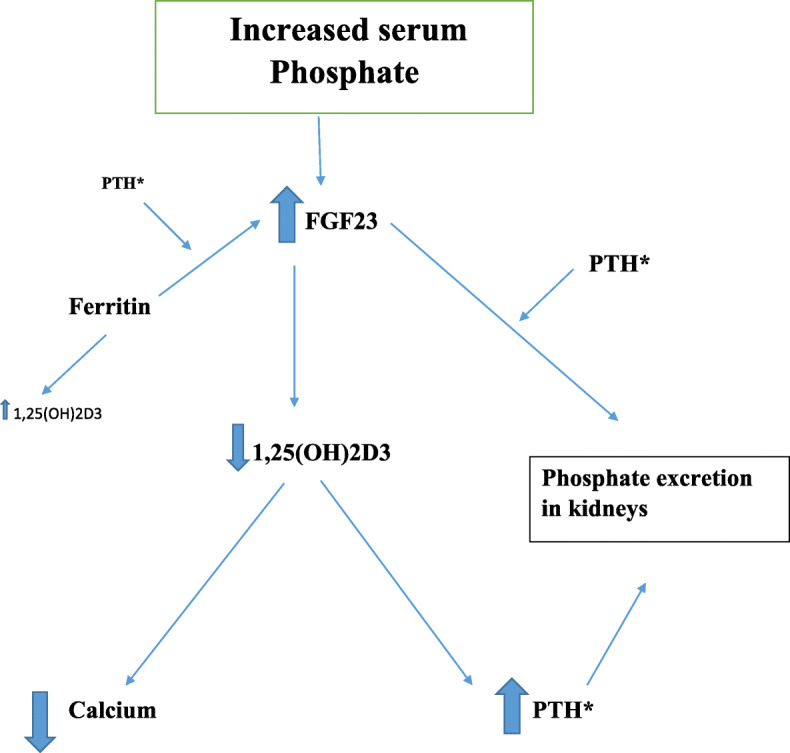
Relationship between serum FGF23, PTH, Phosphorous, PTH and ferritin (*: lack of sufficient PTH in hypoparathyroid patients could interfere within these interactions)

In the present study, a high serum level of FGF23 and 1,25OH2D and a high-normal PTH level in normo-parathyroid controls were observed. Moreover, a strong positive correlation between 1,25(OH)2D3, FGF23 and ferritin levels were detected in the control group. Expectedly in this study, an association between low serum calcium with low serum PTH in patients with hypoparathyroidism was found. In contrast, a high-normal serum calcium and PTH in the control group was observed. This suggests that other factors might be involved in the stimulation of parathyroid secretion. It seems that high ferritin levels in thalassemia patients might have had possible stimulatory effect on PTH secretion in normal parathyroid function, resulting in high-normal serum PTH and calcium (Fig. 2).

Kurtoglu et al. showed a high PTH level in major thalassemia patients more in their first two decades [2]. Additionally, another study on 90 patients with thalassemia showed that more than 25% of them had high-normal levels of PTH and calcium. They also found a significant correlation between ferritin and PTH in these patients [29]. Pawlotsky et al. revealed a positive correlation between serum ferritin and raise in serum PTH 44–68 levels in patients with iron overload syndrome; however, there was no correlation with intact PTH [30]. On the other hand, some patients with thalassemia may develop parathyroid dysfunction at older ages because of iron overload and iron deposition on the parathyroid glands [2]. The iron overload could induce lysosomal and sarcolemmal membrane damage through free radical formation and lipid peroxidation; and causing the destruction of parathyroid glands might be the underlying mechanism [31]. Moreover, cell surface transferrin receptors could play a role in protecting parathyroid glands against inorganic iron [32].

In the present study, we noticed that both case and controls had insufficient 25(OH) D serum levels. Napoli et al. reported serum 25(OH) D deficiency in adult patients with beta thalassemia [33]. In this study, a high normal 1,25(OH)2D3 serum level despite 25(OH) D deficiency was observed in the control group. High normal serum PTH might be a potent factor to enhance alfa-1-hydroxylase activity in these patients. Additionally, this study showed a strong positive correlation between 1,25(OH)2D3 and ferritin levels in the control group, which was not observed in patients with hypoparathyroidism. Therefore, we hypothesized that in case of intact parathyroid function, high ferritin level might enhance 1,25OH2D production through direct stimulation of alfa-1-hydroxylase or indirectly through parathyroid hormone action. Some previous reports showed a significant low level of vitamin D in thalassemia patients, but only few of these studies provided the serum 1,25(OH)2D3 levels of those patients [1, 33]. Wood et al. showed a high serum level of 1,25(OH)2D3 in patients with thalassemia. He suggested that it could occur in spite of primary hyperparathyroidism or upregulation of extra-renal alfa-1 hydroxylase activity [34]. Another study by Dandona et al. showed normal 1,25(OH)2D and PTH concentrations despite vitamin D deficiency in thalassemia patients and claimed for an important role for vitamin D deficiency in the pathogenesis of thalassemia osteopathy [35]. However, a high level of 1,25(OH)2D3 usually does not have its full function in intestinal calcium absorption of thalassemia patients. Moreover, Charoenphandhu et al. found that 1,25(OH)2D3-dependent intestinal calcium absorption was only observed in wild-type mice and not in the beta-thalassemia mice. They concluded that in beta-thalassemia mice, the 1,25(OH)2 D3-dependent intestinal calcium absorption was impaired at the post-transcriptional level, which could lead to the dysregulation of body calcium metabolism and osteopenia [36].

This study revealed a normal serum phosphate level in spite of raised FGF23 and high urinary phosphate loss in the control group. It was suggested that the high serum level of 1,25(OH)2D3 in patients with thalassemia could enhance the intestinal phosphate absorption, which leads to a normal serum phosphate even with high urinary phosphate loss [37]. Another finding of the present study was the high level of serum FGF23 in patients with thalassemia. Two mechanisms could be put forward to explain the increase of serum FGF23 in these patients. The first is the stimulatory effect of PTH or 1,25(OH)2D3 on FGF23 production [38, 39]. In line with this, Moshayoff et al. showed that serum FGF23 levels were increased by PTH administration in both in vivo and in vitro [40]. In addition, one study revealed that PTH had direct and indirect effects through 1,25(OH)2D3 on FGF23 secretion [41]. In the previous studies, the role of Inflammatory Mediators was demonstrated as a potential factor in elevation of FGF23 [19, 20]. However, rise of serum ferritin in transfusion dependent thalassemia is related to the repeated transfusions and red blood cells destruction independent of any inflammatory process. Present study revealed that there was a strong positive correlation between ferritin and FGF23 in our patients with thalassemia, it could be suggesting another possible mechanism by direct stimulatory effect of ferritin on FGF23 secretion, which should be more investigated in future studies.As the result of a strong positive correlation between ferritin and FGF23 in our patients with thalassemia, another possible mechanism could be the direct stimulatory effect of ferritin on FGF23 secretion, which should be further investigated in future studies.

There are controversies about the effects of iron deficiency or iron overload on the serum level of FGF23. Recent studies have shown that iron deficiency could increase the FGF23 degradation, and administration of parenteral iron products, such as ferric carboxy-maltose increases FGF23 level [15, 16, 18]. On the other hand, some studies have demonstrated an association between iron deficiency and increase in serum FGF23, and conversely iron transfusion resulted in the decline of FGF23 level [17]. Iguchi A, et al. showed that Oral iron administration which is used in treatment of iron deficiency anemia could reduce serum FGF23 levels and increase serum PTH levels, despite the phosphate levels remained unchanged. Hence, the iron load could have been the main reason of altered serum FGF23, because the phosphate levels did not change [42]. Another research by Tangngam et al. indicated that the plasma level of FGF23 in the normal controls was significantly higher than the thalassemia group [43].

The present study also showed that despite an increase in the FGF23 serum level in the studied thalassemia patients affected by hypoparathyroidism and hyperphosphatemia, no rise in the urinary phosphate excretion was observed. Recently, a few studies have been performed to evaluate the effect of PTH on FGF23 function in phosphate homeostasis [44]. Yamashita et al. evaluated the changes of FGF23 and its role, in phosphorous homeostasis in patients with transient hypoparathyroidism after thyroidectomy. They showed that FGF23 was increased in hypoparathyroid and hyperphosphatemic state, which was normalized along with serum phosphate stabilization after recovery of parathyroid function. The peak phosphate level always preceded that of FGF23 by several days, suggesting that an elevated phosphate level is the primary stimulus for FGF23 production to maintain serum phosphate homeostasis. This homeostatic regulation of phosphate differs significantly from that of serum calcium changes which corrected fast within several minutes [45]. The present study might suggest that FGF23 was not able to exert its full impact in reducing serum phosphate in the absence of PTH. This could be due to the role of PTH in regulating FGF23 function.

Despite many interesting findings in this study, there were some limitations; first, the present study was descriptive with a small number of patients and not a clinical trial. Since the patients were not from naïve/pre-treatment ones, the analyzed parameters could be altered by the employed treatment. Such therapies still hamper the strength of our conclusion on the pure regulatory feedback existing between iron and mineral hormones. However, wash out from vitamin D and calcium is not ethically or medically acceptable. Although the last guideline for the management of transfusion dependent thalassemia (TDT) recommend that serum ferritin generally correlates with body iron stores, and is relatively easy and inexpensive to determine repeatedly, it was also shown that liver iron content using liver biopsy is the most reliable indicator of body iron load. However, it is invasive and expensive and considered for those with serum ferritin more than 3000 ng/ml [46]. Hence, future clinical trials are suggested on patients with hypoparathyroidism before treatment to investigate the FGF23 functions and its interaction with PTH more accurately, considering tissue iron evaluation e.g. in liver. Moreover, investigating FGF23 and PTH gene expression could help to gain more insight into their physiology and their interaction. Further studies are also suggested to evaluate the role of ferritin on PTH, FGF23 in normal population with and without hypoparathyroidism and also in other genotypes of thalassemia.

## Conclusion

The present study suggested that the rise in FGF23 in patients with thalassemia may be associated with either the stimulating effect of PTH and 1,25(OH)2D3 on FGF23 production, or direct induction effect of ferritin. In addition, we hypothesized that in the case of intact parathyroid function, high ferritin level might enhance 1,25OH2D production possibly through direct stimulation of alfa-1-hydroxylase or indirectly by increasing the parathyroid hormone. Future clinical trials should be conducted on patients with hypoparathyroidism after PTH treatment to investigate the FGF23 functions more accurately.

## Data Availability

The datasets used and/or analyzed during the current study are available from the corresponding author on reasonable request.

## Abbreviations

1,25(OH)2 D: 1,25-dihydroxyvitamin D
25OHD: 25-hydroxy vitamin D
ALP: Alkaline phosphatase
Ca: Calcium
CKD: Chronic kidney diseases
DXA: Dual-energy X-ray absorptiometry
ECLIA: Electrochemiluminescence’s Immunoassay
FE PO_4_: Fractional excretion of phosphorous
FGF23: Fibroblast growth factor 23
PTH: Parathyroid hormone
PO_4_: Phosphorus
SUMS: Shiraz University of Medical Sciences
SPSS: SPSS statistical software
TDT: Transfusion dependent thalassemia
